# A Treatise on Moxa, as Applicable More Especially to Stiff Joints, &c.

**Published:** 1825-04-01

**Authors:** 


*A Treatise on Moxa, as applicable more especially to Stiff Joints: illustrated by Cases and Plates: with some general Observations on Spinal Disease.* By James Boyle, Esq. Surgeon to the Middlesex Infirmary—Author of a Treatise on the Epidemic Cholera of India, &c. Octavo, pp. 168, two plates. London, January, 1825. Price 7*s.* in boards.

This little work is dedicated in respectful language to Dr. William Burnett, Commissioner for the Medical Department of the Navy, and then opens with some introductory observations on the application of *caloric*, as a powerful agent in the treatment of chronic diseases. Mr. Boyle avers, that our Continental brethren, while they have given abundant proofs of the practical advantages of heat, mechanically applied to the human body, have left entirely unexplored the *physiological* action of this powerful material. But we cannot stop at the physiological threshold of the work. The Treatise on Moxa is very short, as far as the history of this remedial agent is concerned—and it was wisely made succinct, as Dr. Dunglison's translation of Baron Larrey's memoir on the subject, supersedes the necessity of any lengthened detail. It will appear from the following quotation, that Mr. Boyle's method of applying the moxa differs from that employed on the Continent.

"In this country *moxa* is known by little more than name—a name too, which, from the impression it conveys, is not likely to recommend it to general notice. Such indeed is the severity with which the operation is performed on the continent, where the remedy is in common use, that the objections held against it in England, both by practitioner and patient, are not much a matter of surprise. The circumstance of deliberately and slowly burning a part till an eschar is formed, must ever bear the semblance of rudeness and barbarity, whatever may be the result of the process. This, however, is not the only objection, another of far greater consequence exists, namely, that *moxa*, when applied to the extent and rapidity of a cautery, is not capable of effecting the same or equally important purposes which a gradual or slow process has the power of doing. This idea was conceived from a physiological consideration of the action of heat on the human body at various temperatures, and gave rise to a modification of practice, in which the author feels borne out by the result of the following extraordinary cases, which, as they offered resistance to every other chirurgical agent, except the one now under discussion, will perhaps be considered the best proof in favour of the remedy, and of the mode of applying it hereafter to be explained." 21.

We shall now proceed to give an account of some of the cases recorded in this volume.

*Case* 1. This was an Officer in His Majesty's service, who, while serving in India, experienced a severe attack of acute rheumatism in the right knee.

"On his arrival in England, his general health had suffered serious deterioration; the muscles of the leg and thigh were much wasted, and the knee was still enormously swollen. It was supposed, that the local affection depended upon a generally deranged state of his health, and that by improving the latter, the former would subside of itself; so physicians of eminence were consulted, till at length good health was re-established; but without effecting the slightest improvement in the local disorder. The case being now altogether surgical, he had the advice of those men, who in reputation justly stand at the head of the profession: each pursued his particular plan of practice, and each in turn pronounced the case one not to be cured. In addition to all the common, and more probable means of success, *Mahommed*, of Brighton's, shampooing and medicated baths were tried. In this way two years were passed; the chances of recovery being naturally diminished in proportion to the duration of the complaint: last of all, he was advised to relinquish every idea of public service—an advice which distressed him the more, as he was possessed of singular zeal and a laudable ambition to rise in his profession. Such were the outlines of this gentleman's case, and such were his prospects of recovery!

"When consulted, I found the knee to be greatly enlarged, painful, and hot to the touch; the enlargement being the more conspicuous, in consequence of great wasting both above and below the joint. There was scarcely any action of the leg upon the thigh, and the patella had no apparent motion, being bound down, I supposed, by thickening and condensation of the cellular membrane with which it was surrounded. On each side of the ligamentum patellæ, immediately under the inferior margin of the patella, a hard mass of substance, about the size of a finger, and having a cartilaginous feel, greatly contributed to the fixed state of that bone; limiting the sphere of its natural action, and rendering its motion impossible. It was believed that a thickening of the ligaments, but particularly of the capsular, existed; and from the great degree of heat, as well as much sensitiveness, it was concluded that deep-seated inflammation was still going forward. With this impression, twenty four leeches were applied, and the bleeding kept up by hot fomentations. On the second day following, the heat of the part being still above the natural standard, twenty more leeches were had recourse to; the fomentations were again used, and on this occasion a large bread poultice was placed round the knee. After this, evaporating lotions were persisted in, till the temperature of the affected part was reduced to par. Fearing now a sudden reflux of blood to the numerous enlarged vessels surrounding the knee, small doses of digitalis were administered: this appeared to answer the purpose effectually, so the application of *moxa* next became the remedy for consideration.

"A small quantity of the *Chinese moxa* having been procured, about the size of a hazel nut, compressed between a pair of common dressing forceps, was lighted and held as near the part as could well be borne, till it entirely burned away; and this was repeated three or four times on each occasion of its application, which at first was three times a week, varying a little, the part over which it was placed at each repetition. To effect motion of the patella being an object of great importance, attention was more particularly directed to that. In general, therefore, the circumference of the patella was divided into four portions, which were considered the most favourable for the action of the material under consideration.

"After this apparent ordeal, which, in truth, was by no means painful, the kneee was rubbed for fifteen minutes with camphorated oil; a flannel bandage was then passed with moderate tightness, for the purpose of keeping up whatever new action in the absorbents and veins the *moxa* might have induced. Finally, manual efforts were used to extend the limb; supposing that much of the stiffness depended upon a rigidly contracted state of the fibres of the flexor muscles. In a few days it was satisfactory to observe the advantages of this system; for although the improvement was, at first, so slow as to be scarcely perceptible to the eye, we had assured ourselves of its reality, by applying a scale in the ham, and marking it up to the point gained. The circumference of the knee was soon reduced; but the thickening of the cellular substance in the neighbourhood of the inferior portion of the patella was so indolent, that caustic issues were deemed advisable: this, however, did little except interfere with the more successful course, which had been previously commenced, and was consequently discontinued. Having expended all the foreign *moxa*, which could be procured in London, recourse was had to the expedient of substituting a piece of folded calico, which, being lighted, was kept burning by means of a pair of bellows, after the manner in which this remedy is usually applied in the Parisian hospitals; but this being objectionable, on account of the intense heat which it caused, as well as a most acrid and offensive smoke, a composition resembling the Chinese *moxa*, was obtained from Paris: this secured the preference, and being easily imitated, a similar preparation was continued."* 29

Attention was directed to every part of the knee in which pain was felt, until the joint had acquired nearly as much free-

"* This preparation is made by dissolving one ounce of nitrate of potass in a quart of water, and therein saturating a quantity of fine cotton wadding, which being dried and formed into small parcels, is enclosed within paper cylinders, about half-an-inch in diameter, and one inch in height. It burns in an extremely slow and gentle manner; and only requires being kept dry, to be fit for use at all times. I do not know any substance so well calculated to fulfil the purposes for which this is employed."

dom as in the natural state. By this time the muscles of the leg and thigh had filled out—flexion and extension were almost complete—" in fact, the parts surrounding the knee presented nearly their proportionate form and pliability:—finally, walking, dancing, riding, and the hot baths of Brighton, were practised as a wind up to the cure." This gentleman is now employed in the public service of his country, and enjoys perfect health.

We have given the above case more at length than we are accustomed, because it shews what may be done by perseverance in manual and external measures, in cases of stiffened and otherwise diseased joints.

*Case* 2. This was a lady who had had acute rheumatism also, affecting more particularly the right wrist, which was stiffened, the muscles weakened, and pronation and supination entirely prevented. Moxas were applied on the muscles of pronation and supination, and shampooing diligently employed, with frictions of camphorated oil. " In the course of three days an amendment was perceptible, and at the expiration of fifteen days, the powers of the arm were almost perfect."

We can only make room for one more case, which was that of a lady of high rank, and where, we have reason to believe, the success was very striking, and the advantage to the author not inconsiderable.

*Case* 3. " A lady of high rank had an arm lame for eight years—the following are the outlines which were given ;—

" After *accouchement*, a train of symptoms and circumstances, the description of which would be superfluous here, preceded and gave rise to violent inflammation of the arm ; the back of the hand, the wrist, and the internal part of the arm, near the elbow suffered more particularly.

" On examination, I found the hand to be bent inwards, upon the forearm, forming an arc of about an inch and a half in depth. And, on passing the fingers along the forearm, in the direction of the *flexor sublimis perforatus*, the centre of that muscle was found to be much contracted, having formed several distinct indurated projections ; at its origin, there was a mass of contracted muscle. The elbow joint was perfect, but that of the wrist admitted not of the slightest perceptible motion. All the fingers of this hand were more or less stiff ; the middle finger was completely so, and was frequently a cause of great annoyance, by striking against hard bodies, such as doors, tables, chairs, parts of the carriage in getting into it, &c. &c. The power of extending the fingers one from another was greatly limited. From these circumstances, her harp, which was formerly to this lady, a source of the greatest delight, had stood quite idle during the long period of the above misfortune; the *piano*, which was less difficult, though less a favourite than its companion, became a substitute in consequence; and was surprisingly executed upon by the increased powers which practice gave to the left hand. From the advantages of wealth and liberality, this case had not suffered for want of numerous medical opinions, and the full trial of varied means of cure in this country, as well as on the Continent, where, finally, it was given over as a hopeless case, and had not since that period, a space of six years, undergone any treatment.

"The practice commenced by applying daily three or four *moxa* cylinders at regular distances, in the direction of the *flexor sublimis perforatus*, followed by friction with camphorated oil, and also mechanical extension. In the short period of a week, motion was perceptible at the wrist, and the masses of thickened fibres from this time began to disperse. Much friction and shampooing were now had recourse to, as well as the application of the moxa to all the joints of the fingers, and the whole course of the above described muscle. After conducting the practice in this manner for some weeks, cork splints, neatly padded and covered, were brought forward as an additional auxiliary. The other processes having been gone through, they were applied daily, and allowed to remain on for the space of half an hour. These splints had the peculiar advantages of accommodating themselves, in some measure, to the resisting force of the muscles opposed to them; and being secured by straps, easily admitted of an increase or diminution of the extending power. In this manner the flexor muscles were soon greatly relaxed, and the fingers acquired a proportionate degree of suppleness. The action of the wrist, however, was still beyond the control of the muscles; a certain degree of force being yet necessary to effect the motion which had been thus acquired." 38.

It was now supposed that ossific union between the articulating extremities of the ulna and radius and the carpal bones had taken place; hence further attempts to *cure* this joint were laid aside, and undivided attention was directed to the fingers, and those muscles under whose more immediate influence they are, in the healthy state. Moxa was applied to the respective joints of the fingers, followed by friction and shampooing—which system was persevered in for five months, at the end of which time, "every finger had regained its long-lost pliability—the arm had restored to it again almost all its former symmetry, and this lady had the gratification of being able, once more to resume her former favourite amusement in music." 39.

For many other cases, we must refer to the volume itself. The work concludes with some general but judicious observations on spinal diseases. But as we have occupied an unusual portion of our journal lately with this subject, we cannot enter on it in this place.

Respecting the powers of moxa, we can say nothing from personal observation; but we have seen so much good result from friction and shampooing, that we have little doubt that moxa, in conjunction with these means, may become very successful, in skilful hands. Mr. Boyle is deserving of thanks for laying the result of his experience before the public, without reserve, or the least attempt to conceal any part of the process employed by him on different occasions.

---

